# Supplementary material for: Influenza hemagglutinin subtypes have different sequence constraints despite sharing extremely similar structures
Source: bioRxiv. 2026 Jan 6:2026.01.05.697808. Preprint. [Version 1] doi: 10.64898/2026.01.05.697808 (PMC12803193; doi:10.64898/2026.01.05.697808)
Supplement: Supplementary file 1 [file NIHPP2026.01.05.697808v1-supplement-1.pdf]

bioRxiv preprint doi: https://doi.org/10.64898/2026.01.05.697808; this version posted January 6, 2026. The copyright holder for this preprint (which was not certified by peer review) is the author/funder, who has granted bioRxiv a license to display the preprint in perpetuity. It is made available under aCC-BY 4.0 International license.

Supplemental figures

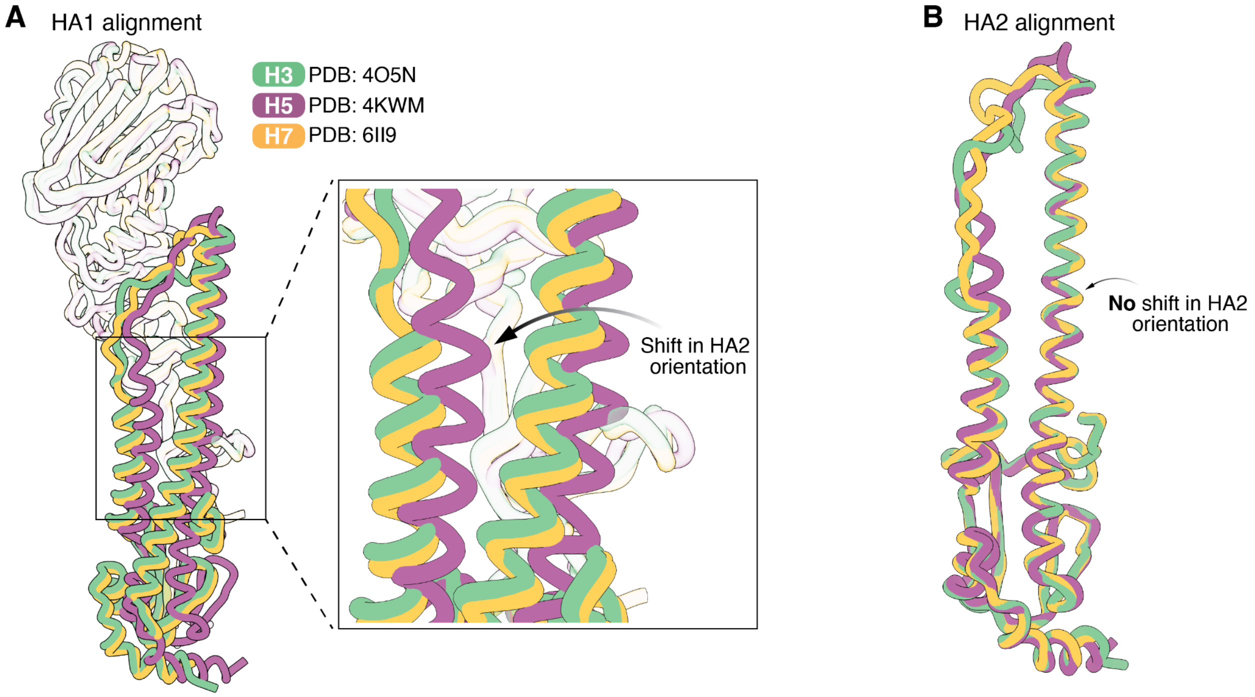

Figure S1 | The relative orientations of HA1 and HA2 are shifted in H5 HA relative to H3 and H7 HAs.

A) Structural alignment of the HA1 domain (same as left structure in **Fig. 1C**) with the HA2 domains from H3 (green), H5 (purple), and H7 (orange) HAs. Due to a tilt in the orientation of the HA1 and HA2 domains, the HA2 domain of H5 becomes shifted relative to where the HA2 domains of H3 and H7 are located. **B)** Aligning the HA2 domain alone (same as right structure in **Fig. 1C**) reveals the folds in HA2 are actually highly conserved across H3, H5, and H7.

bioRxiv preprint doi: https://doi.org/10.64898/2026.01.05.697808; this version posted January 6, 2026. The copyright holder for this preprint (which was not certified by peer review) is the author/funder, who has granted bioRxiv a license to display the preprint in perpetuity. It is made available under aCC-BY 4.0 International license.

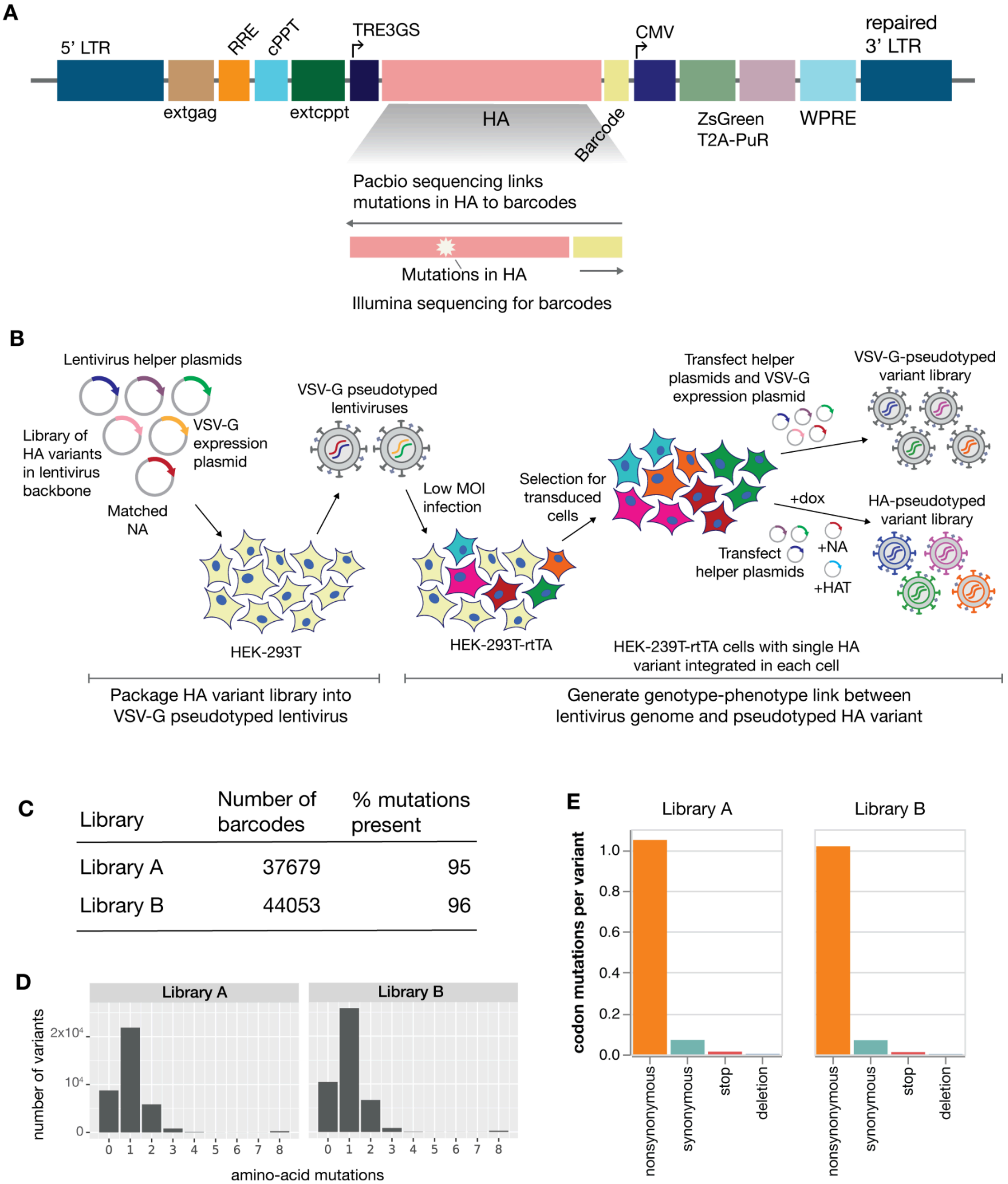

Figure S2 | Overview of pseudovirus deep mutational scanning of H7 HA

A) The pseudovirus deep mutational scanning uses a lentiviral backbone encoding the HA protein followed by an identifying 16-nucleotide barcode under the control of an inducible

bioRxiv preprint doi: https://doi.org/10.64898/2026.01.05.697808; this version posted January 6, 2026. The copyright holder for this preprint (which was not certified by peer review) is the author/funder, who has granted bioRxiv a license to display the preprint in perpetuity. It is made available under aCC-BY 4.0 International license.

promoter. The lentiviral backbone has a full 3′-LTR so that integrated proviruses can be reactivated by transfection of appropriate helper plasmids. **B)** Schematic of pseudovirus deep mutational scanning experiments. We create a library of mutants of the HA in the context of the barcoded-HA expressing lentiviral backbone plasmid. Cells are transfected with this HA-encoding lentiviral backbone along with helper plasmids encoding the other proteins needed to produce lentiviral particles (Gag-Pol, Tat, Rev), a plasmid encoding the matched N9 NA, and a plasmid encoding VSV-G. The resulting lentiviral particles, which lack a genotype-phenotype link and express VSV-G on their surface, are the infected into rtTA expressing 293T cells at low multiplicity of infection, and transduced cells are infected so that each cell encode a unique HA mutant as a provirus in its genome. These cells are then transfected with the helper plasmids and NA-encoding plasmids to produce pseudoviruses that express unique HA mutants. Cells are also transfected with VSV-G to produce control pseudoviruses that are not dependent on HA to infect cells. To measure cell entry of each HA variant, we use sequencing to compare the efficiency of that barcoded lentiviral particle at entering cells in the presence or absence of VSV-G (particles always enter cells when VSV-G is expressed, but otherwise require a functional HA for cell entry). Note that these lentiviral particles encode no proteins other than HA, and so are not capable of undergoing multicycle replication and so provide a safe way to study the effects of mutations to HA. **C)** Number of uniquely barcoded HA variants and the fraction of all possible HA ectodomain amino-acid mutations that are present in at least one barcoded variant in each of the two independent H7 HA pseudovirus libraries. **D)** Distribution of amino-acid mutations per barcoded HA variant in each library. **E)** Average number of nonsynonymous, synonymous, stop-codon, and deletion mutations per barcoded HA variant.

bioRxiv preprint doi: https://doi.org/10.64898/2026.01.05.697808; this version posted January 6, 2026. The copyright holder for this preprint (which was not certified by peer review) is the author/funder, who has granted bioRxiv a license to display the preprint in perpetuity. It is made available under aCC-BY 4.0 International license.

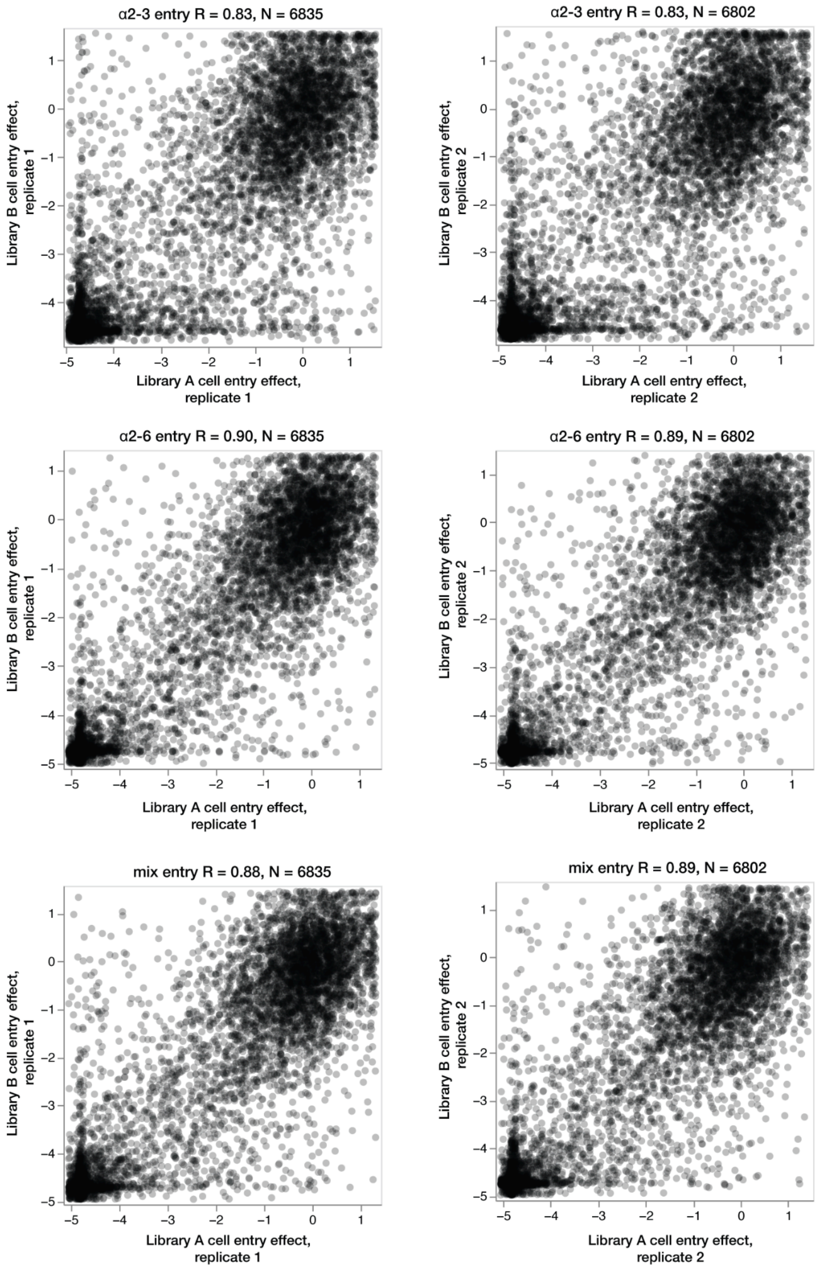

Figure S3 | H7 HA cell entry effects measured using independent deep mutational scanning libraries are highly correlated

Correlation of the effects of H7 HA mutations on cell entry as measured with independent pseudovirus libraries on 293 cells expressing α2-3 linked sialic acids (top row), α2-6 linked sialic acids (middle row), or a mix of both cells (bottom row). Each point represents the effect of a mutation measured in each replicate. For each of the two libraries, two technical replicates were performed. Each panel shows the correlations between two independent libraries (Library A and B) for each technical replicate (replicate 1 or 2). R is the Pearson correlation and N is the number of mutations measured in both pairs of measurements.

bioRxiv preprint doi: https://doi.org/10.64898/2026.01.05.697808; this version posted January 6, 2026. The copyright holder for this preprint (which was not certified by peer review) is the author/funder, who has granted bioRxiv a license to display the preprint in perpetuity. It is made available under aCC-BY 4.0 International license.

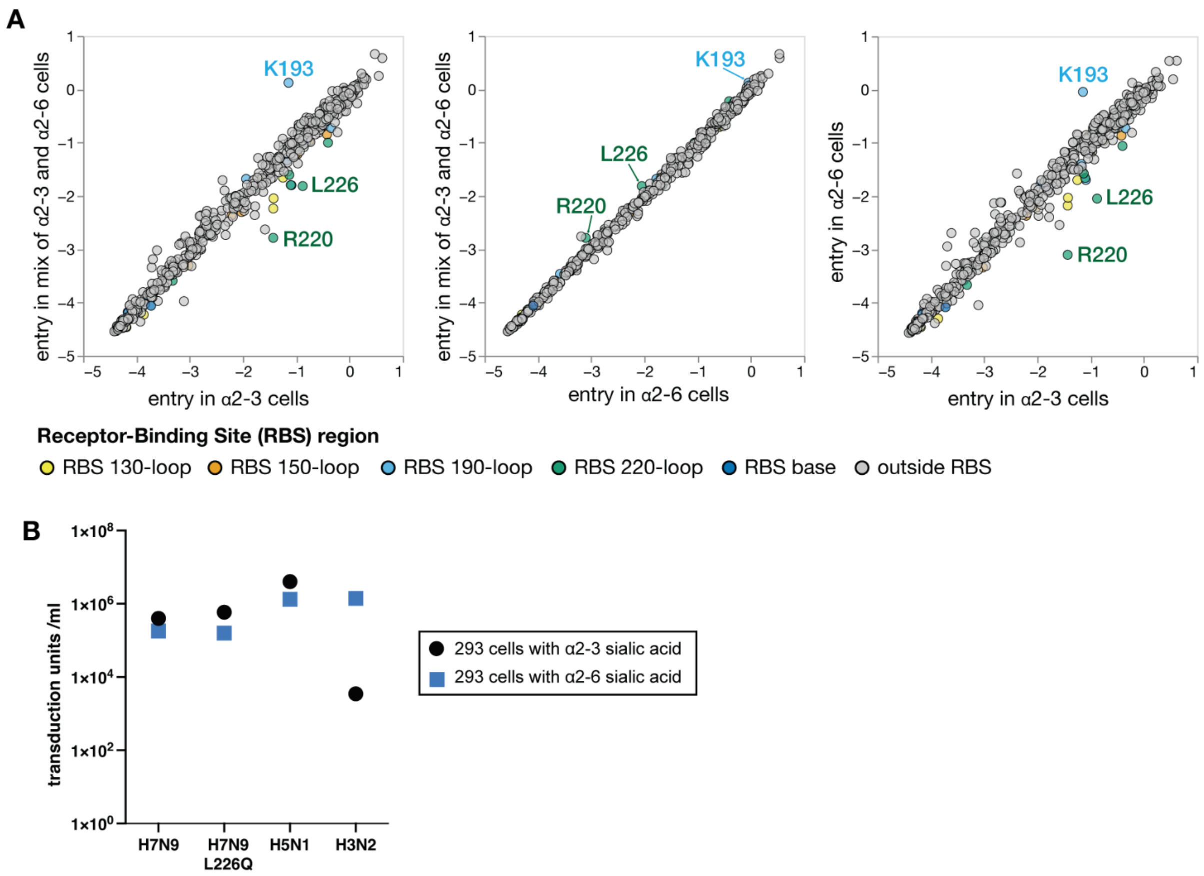

Figure S4 | Effects of mutations to the H7 HA on entry in 293 cells expressing only α2-6 or only α2-3 linked sialic acids

A) Scatter plots showing the average effects of mutations at each site on entry into 293 cells expressing only *α*2-3 linked sialic acids, only *α*2-6 linked sialic acids, or an equal mix of the two cells. Sites are colored by whether they are part of the receptor-binding site (RBS). See https://dms-vep.org/Flu_H7_Anhui13_DMS/cell_entry.html for interactive versions of these plots. **B)** Titers on 293 cells expressing only *α*2-3 linked sialic acids or only *α*2-6 linked sialic acids of lentiviral particles pseudotyped with the HA and NA of A/Anhui/1/2013 (H7N9) strain, the same H7N9 HA and NA but with the L226Q mutation in HA, the HA and NA of the avian A/American Wigeon/South Carolina/USDA-000345-001/2021 (H5N1) strain, or the HA and NA of the human A/Massachusetts/18/2022 (H3N2) strain.

bioRxiv preprint doi: https://doi.org/10.64898/2026.01.05.697808; this version posted January 6, 2026. The copyright holder for this preprint (which was not certified by peer review) is the author/funder, who has granted bioRxiv a license to display the preprint in perpetuity. It is made available under aCC-BY 4.0 International license.

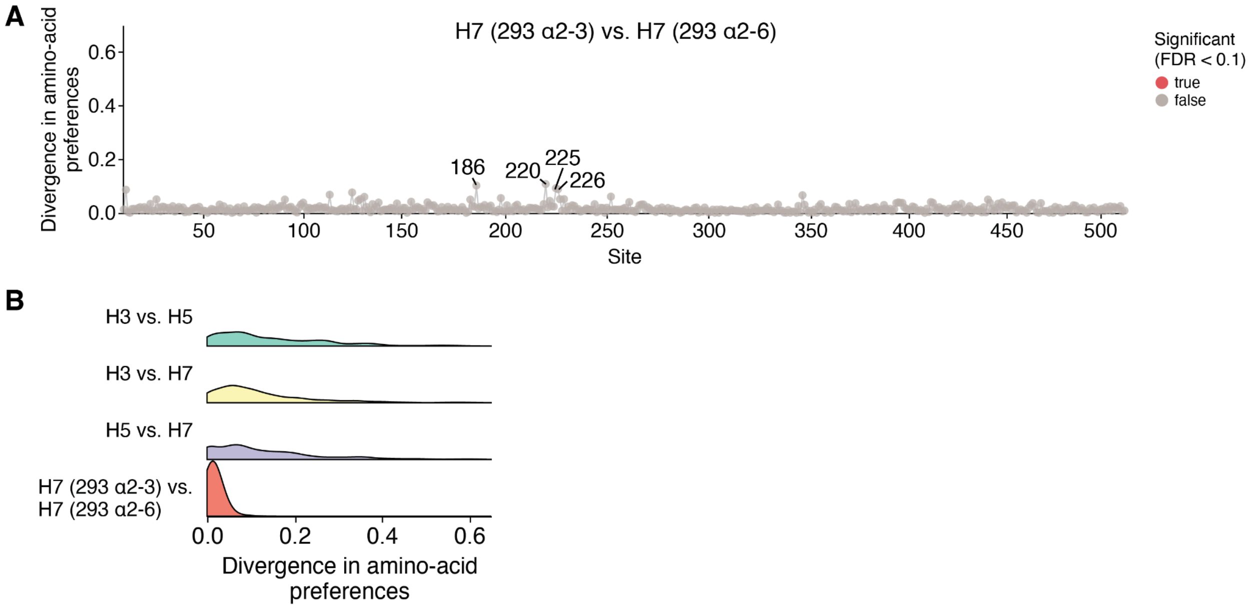

Figure S5 | Divergence in H7 HA amino-acid preferences for entry into 293 cells expressing α2-3 versus α2-6 linked sialic acids is minimal compared to the divergence between HA subtypes.

A) Divergence in amino-acid preferences at each site of the H7 HA as measured in 293 cells expressing only α2-3 versus only α2-6 linked sialic acids. Note that the divergence at all sites is low compared to the cross HA-subtype comparisons in **Fig. 3C**, and no sites are significantly diverged (false discovery rate < 0.1). The largest differences are at sites like 186, 220, 225, and 226, which are important for receptor binding. **B)** Distributions of divergence in amino-acid preferences for all sites between subtypes compared to the distribution of divergence in H7 HA for measurement made using 293 cells expressing entry only α2-3 versus only α2-6 linked sialic acids.

bioRxiv preprint doi: https://doi.org/10.64898/2026.01.05.697808; this version posted January 6, 2026. The copyright holder for this preprint (which was not certified by peer review) is the author/funder, who has granted bioRxiv a license to display the preprint in perpetuity. It is made available under aCC-BY 4.0 International license.

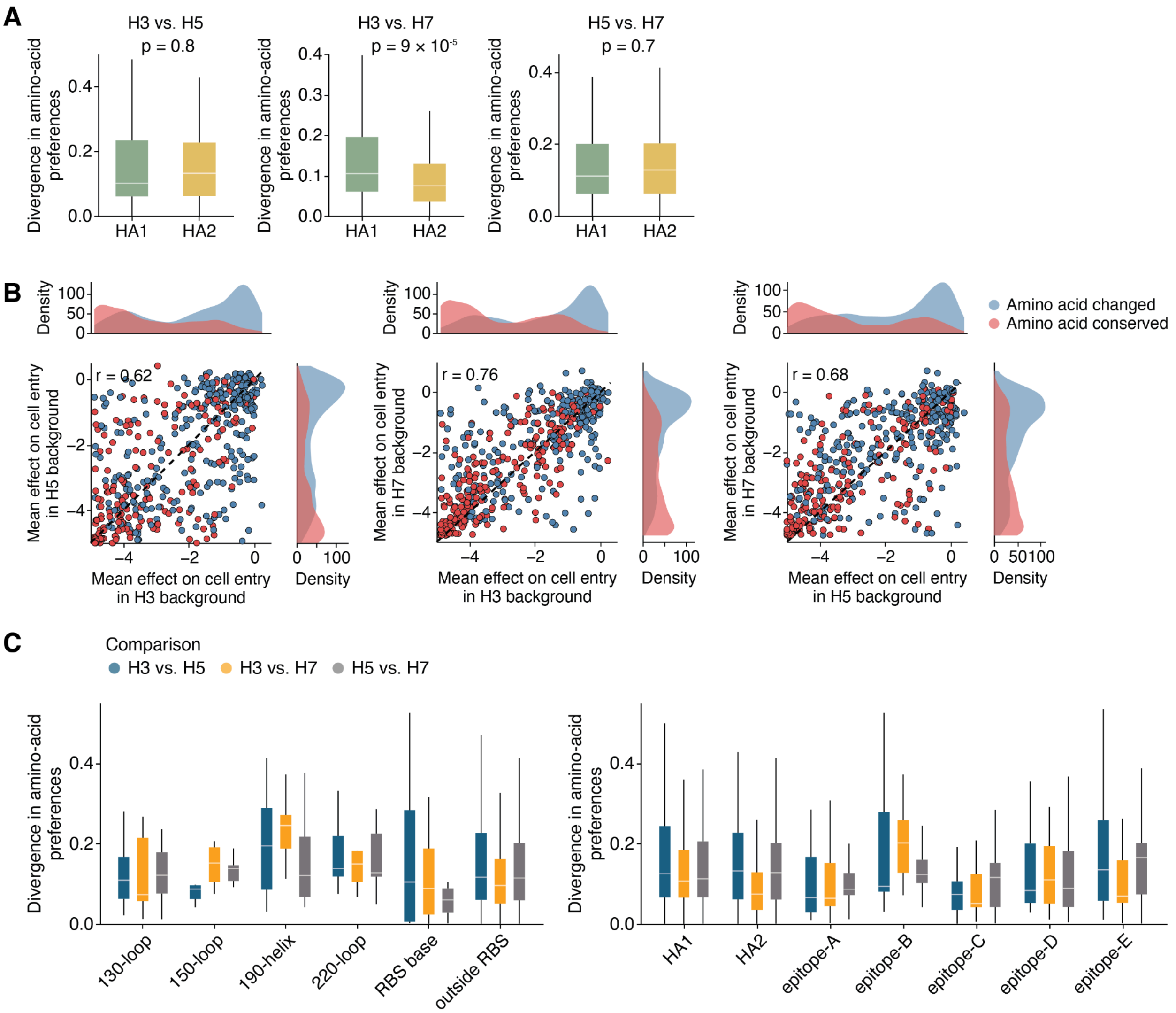

Figure S6 | Divergence in amino-acid preferences for different domains or regions of HA.

A) Distributions of divergence in amino-acid preferences between HA subtypes for sites in the HA1 or HA2 domains of HA. In H3-H7, HA2 sites show significantly lower divergence compared to HA1. **B)** Correlation of the mean effect of mutations at each site between each pair of HA subtypes. Sites where the wildtype amino-acid identity differs between subtypes are colored blue, while sites with conserved wildtype amino-acid identities are red. **C)** Distributions of divergence in the amino-acid preferences by receptor binding pocket region (left) and antigenic region (right) across each HA comparison.

bioRxiv preprint doi: https://doi.org/10.64898/2026.01.05.697808; this version posted January 6, 2026. The copyright holder for this preprint (which was not certified by peer review) is the author/funder, who has granted bioRxiv a license to display the preprint in perpetuity. It is made available under aCC-BY 4.0 International license.

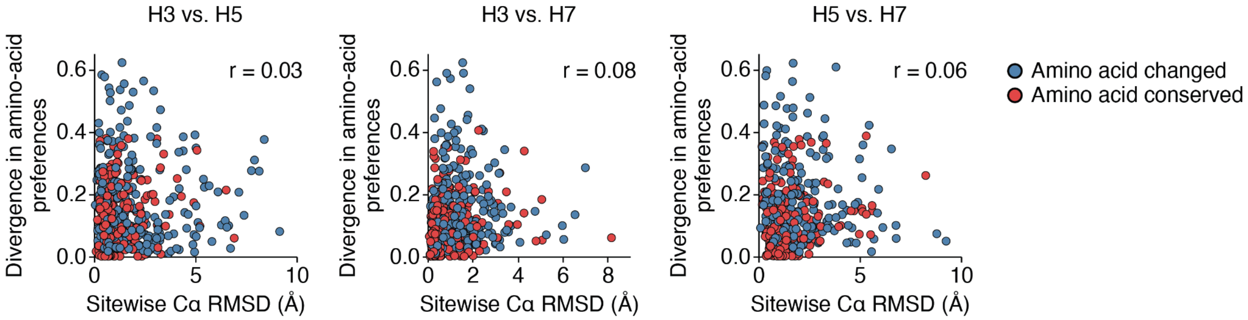

Figure S7 | Structural deviation is a poor predictor of divergence in amino-acid preferences at a site.

Correlation between divergence in amino-acid preferences and the Cα root mean square deviation at each site in the structurally aligned backbones of HA1 and HA2 across pairwise HA comparisons. Sites where the wildtype amino acids changed between subtypes are colored blue, while conserved sites are red.

bioRxiv preprint doi: https://doi.org/10.64898/2026.01.05.697808; this version posted January 6, 2026. The copyright holder for this preprint (which was not certified by peer review) is the author/funder, who has granted bioRxiv a license to display the preprint in perpetuity. It is made available under aCC-BY 4.0 International license.

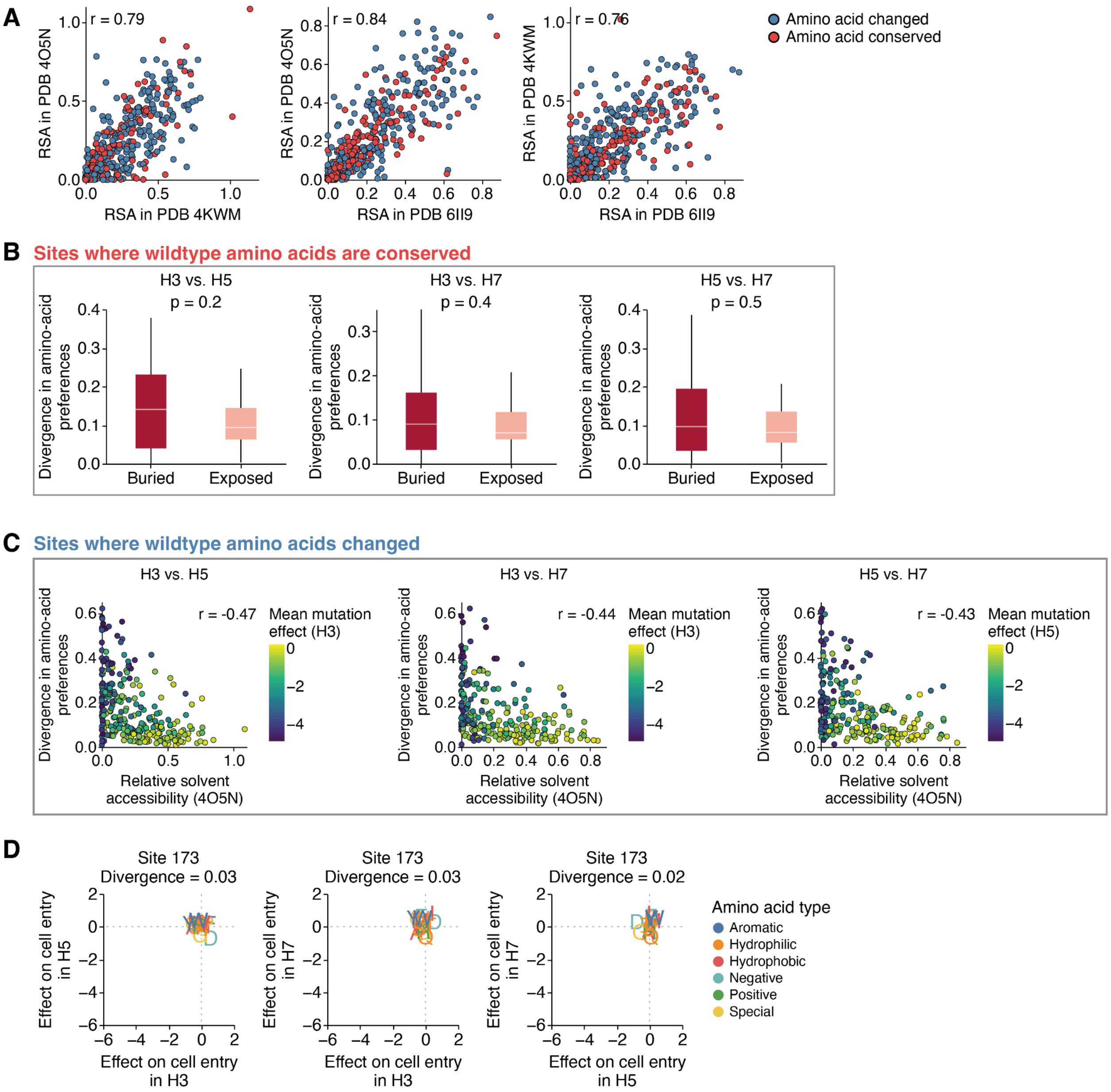

Figure S8 | Surface exposed sites are more mutationally tolerant and display less divergent amino acid preferences across HAs.

A) Correlation between relative solvent accessibility values at structurally aligned sites between HAs. These values quantify how exposed or buried a site is, and the correlation is strong ($r >$ 0.75) because the HA structures are conserved. PDB accessions are 4O5N (H3), 4KWM (H5), 6II9 and (H7). **B)** Among sites where the wildtype amino-acid identity is conserved (red boxplots in **Fig. 4A**), there is no significant difference in divergence in amino-acid preferences at buried versus exposed sites. **C)** Among sites where the wildtype amino-acid identity changed (blue boxplots in **Fig. 4A**), there is a negative correlation between divergence in amino-acid preferences and relative solvent accessibility. Sites are colored by mean mutation effect on entry in the indicated HA background, showing that sites that are more exposed tend to tolerate

bioRxiv preprint doi: https://doi.org/10.64898/2026.01.05.697808; this version posted January 6, 2026. The copyright holder for this preprint (which was not certified by peer review) is the author/funder, who has granted bioRxiv a license to display the preprint in perpetuity. It is made available under aCC-BY 4.0 International license.

mutations better. **D)** Correlations of effects of mutations on cell entry between HAs at the exposed site 173, showing how all measured mutations are tolerated across HAs at this site. Two-sided Mann-Whitney-U test was used for all significance testing.
